# Mapping of machine learning approaches for description, prediction, and causal inference in the social and health sciences

**DOI:** 10.1126/sciadv.abk1942

**Published:** 2022-10-19

**Authors:** Anja K. Leist, Matthias Klee, Jung Hyun Kim, David H. Rehkopf, Stéphane P. A. Bordas, Graciela Muniz-Terrera, Sara Wade

**Affiliations:** ^1^ Department of Social Sciences, Institute for Research on Socio-Economic Inequality (IRSEI), University of Luxembourg, Esch-sur-Alzette, Luxembourg.; ^2^ Department of Epidemiology and Population Health, Stanford University, Palo Alto, CA, USA.; ^3^ Department of Engineering, University of Luxembourg, Esch-sur-Alzette, Luxembourg.; ^4^ Centre for Dementia Prevention, University of Edinburgh, Edinburgh, UK.; ^5^ Ohio University, Athens, OH, USA.; ^6^ School of Mathematics, University of Edinburgh, Edinburgh, UK.

## Abstract

Machine learning (ML) methodology used in the social and health sciences needs to fit the intended research purposes of description, prediction, or causal inference. This paper provides a comprehensive, systematic meta-mapping of research questions in the social and health sciences to appropriate ML approaches by incorporating the necessary requirements to statistical analysis in these disciplines. We map the established classification into description, prediction, counterfactual prediction, and causal structural learning to common research goals, such as estimating prevalence of adverse social or health outcomes, predicting the risk of an event, and identifying risk factors or causes of adverse outcomes, and explain common ML performance metrics. Such mapping may help to fully exploit the benefits of ML while considering domain-specific aspects relevant to the social and health sciences and hopefully contribute to the acceleration of the uptake of ML applications to advance both basic and applied social and health sciences research.

## Teaser

ML methods are comprehensively reviewed for their optimal use in epidemiology, demography, psychology, health care, and economics.

## INTRODUCTION

Compared to many traditional statistical methods and with increasing availability of large datasets of relevance to the social and health sciences, machine learning (ML) methods have the potential to considerably improve aspects of empirical analysis. This includes advances in prediction, by fast processing of large amounts of data; in detecting nonlinear and higher-order relationships between exposures and confounders; and in improving accuracy of prediction. However, uptake of ML approaches in social and health research, spanning from sociology, psychology, and economics to social and clinical epidemiology and public health, has been rather slow and remains fragmented to this date. We argue that this is, in part, due to a lack of communication between the disciplines, the limited incorporating of domain knowledge into analytical approaches in the social and health sciences, and a lack of accessible overviews of ML approaches fitting the research goals in the social and health sciences.

The aims of this paper are to provide a high-level, nontechnical toolbox of ML approaches through the systematic mapping of research goals in the social and health sciences to appropriate ML methods; explain common metrics in ML; and point researchers to solutions to common problems in ML modeling. Our review focuses on research questions that involve datasets with human participants as research units and the analysis of clinically assessed or self-reported variables. In most studies in the social and health sciences using ML, we present here models that are trained on static datasets, that is, models are not continuously processing new data but rely on finite datasets from cohort studies or surveys after the end of data collection and cleaning.

Our review should be seen as complementary to introduction papers to ML in the fields of epidemiology and health research (*1*), psychology (*2*), and economics (*3*). For general introductions to statistical learning, interested readers are referred to excellent textbooks on these approaches (*4*, *5*).

The remainder of the review is organized as follows: The “Mapping research purposes in the social and health sciences to ML tasks” section outlines the main task of mapping research purposes in the social and health sciences to appropriate ML approaches. The “Basics of ML” section covers the basics of ML, specifically traditional ML categorizations, data preparation, model building, and “real-world” applications of ML. The next sections describe the mapping of ML approaches to research purposes of description, prediction, and causal inference, mapping appropriate ML methods and giving empirical examples. The “ML performance metrics” section gives an overview of ML performance metrics. The “Looking forward” section closes with an outlook.

## MAPPING RESEARCH PURPOSES IN THE SOCIAL AND HEALTH SCIENCES TO ML TASKS

Common research purposes in the social and health sciences can be categorized, in a nutshell, as researchers’ intentions to (i) describe phenomena, (ii) predict social or health outcomes, and (iii) find causes of and possibilities to intervene to improve these outcomes. We will, over the course of this review, present in more detail specific research questions related to description, prediction, and causal inference (*6*), even if not all research questions allow these strict distinctions. We will map these research questions to appropriate ML methods, using empirical studies as illustration where possible.

Methods summarized as ML in this review represent different traditions of data analysis, e.g., inferential statistics, statistical learning, and computational sciences. Their common denominator is the ability to process large amounts of data, while model building and model selection decisions are more driven by the data structure (data-driven) than in traditional inferential statistics.

Both statistical and domain knowledge are relevant when applying ML to research questions in the social and health sciences. Exploratory data analysis (*7*) is used to summarize and visualize the main characteristics of the data and is an important first step in the data analysis and ML pipeline. However, agnostic data exploration, that is, data analysis without consideration of domain knowledge, will alone provide fewer insights in most cases. Some examples of the importance of domain knowledge in the social and health sciences are as follows:

1) The preference of continuous variables in decision tree and other ML algorithms may lead to overvaluing age as a predictor when, in reality, age is much less strictly bound to social or health phenomena (e.g., strong heterogeneity in cognitive performance across the life course).

2) Missing data may be identified in data-driven analysis as meaningful information (and it often is), which will need, however, to be contextualized with regard to the population observed, as well as exposures and outcomes of interest.

3) Categories identified as relevant in data-driven analysis may not be equally meaningful in conceptual terms, e.g., the education-related category “other” or “none of the above” may reflect untypically low educational levels or educational degrees obtained abroad, which again need to be contextualized.

4) Determinant-outcome associations may be biased because of systematic differences in behavior (and survey participation) of individuals with different sociodemographic and socioeconomic profiles, highly relevant in the research on the social determinants of health.

We note that the last example often signifies injustices in health care, when data inequality and structural discrimination of minority populations may lead to discriminating ML algorithms with potential to further aggravate health inequalities (*8*). While some of the ML approaches presented here require more domain knowledge than others, particularly ML for causal inference, we argue that, in all research questions in the social and health sciences, substantial domain knowledge is necessary to meaningfully contribute to the field and that this is a prerequisite to interpretability (*9*). While computational fields have traditionally emphasized improvements in prediction (and statistical knowledge), social and health sciences have often prioritized explanation (and domain knowledge) (*2*); this review aims to show that we do need both to advance scientific knowledge in the social and health sciences.

There is a need to establish a fluid dialog between researchers from the social and health sciences and methodologically trained researchers to avoid “rediscovering the wheel.” ML researchers may lack domain knowledge and overlook features of data that have been previously found to be highly relevant in the social and health sciences (e.g., oversimplification of recoding of some variables when integrating datasets). In contrast, researchers in the social and health sciences may not be aware of the complex mathematics and statistics behind the algorithms and the fast-progressing developments of improving ML methods. In general, from our own experience, we recommend collaborations across disciplines by inviting data science and ML experts to do research in the social and health sciences and hope that the mapping presented here will facilitate mutual understanding of the different disciplines.

## BASICS OF ML

Before moving to ML for description, prediction, and causal inference, we provide a short overview of ML, starting with (i) the traditional categorization of ML approaches, (ii) considerations on data preparation, (iii) model building, and (iv) trustworthiness in real-world applications of ML.

### Traditional categorization of ML approaches

From a data science perspective, most ML approaches can be categorized into the three main branches of unsupervised learning, supervised learning, and reinforcement learning:

1) Unsupervised learning is an umbrella term for algorithms that learn patterns from unlabeled data, that is, variables that are not tagged by a human. For instance, unsupervised learning will group data instances on the basis of similarity.

2) Supervised learning comprises algorithms that learn a function, which maps an input to an output, by using labeled data, that is, the values of the categories of the outcome variable are assigned meaningful tags or labels. Input would, in the social and health sciences, be termed predictors, independent variables or exposures, and covariates; output would be termed outcomes or dependent variables. Supervised learning requires labeled training data and can be validated in a labeled test dataset. We will present neural networks (often called artificial neural networks) as an example of a predictive algorithm and Bayesian Additive Regression Trees (BART) as an example of ML for causal inference. Penalized regressions such as LASSO (least absolute shrinkage and selection operator) condense the number of variables in the model on the basis of their predictive ability, often helping to arrive at both parsimonious and well-performing models.

3) Reinforcement learning is concerned with an intelligent agent taking decisions to an environment and improving on the basis of the notion of cumulative reward, i.e., the agent will vary and optimize the input on the basis of the feedback from the environment. Reinforcement learning can be applied in contexts where data generation, that is, manipulation of a treatment variable (so called A/B testing) under the control of other features (covariates), is possible.

As a learning technique, transfer learning uses multiple data sources to learn knowledge from one dataset and transfer this knowledge to another dataset from a different population, and potentially even on a different outcome (*10*). Transfer learning will thus transfer learned features from one situation to another (congruent) situation, thereby identifying patterns and behaviors common to a variety of situations. While often used with labeled data and thus mentioned as an approach specific to supervised ML, transfer learning approaches have also been developed, for example, in unsupervised learning and for image recognition and other applications not covered in this review. We will present transfer learning as a proposal to reduce fairness violations in the presence of data inequality (*11*).

### Data preparation

Not unlike in traditional statistics, ML requires careful data preparation. In the following, we discuss aspects specific to ML related to (i) data requirements, (ii) feature selection, and (iii) feature engineering. The latter two are concerned with preparing exposure and outcome variables in a way the algorithm understands (*1*, *12*).

#### Data requirements for ML

Most ML approaches presented here will require large(r) datasets than traditional modeling for the models to outperform traditional modeling in datasets that were not used for model training. Some aspects of the data should be available at a larger quantity, such as time points, variables, or individuals. The rule of thumb would be to have several 10,000 data points available, although this depends on the data and ML method, and some applications have used very small datasets for exploratory analyses (*1*). As discussed later, the number of data points required depends on the trade-off of the data dimensionality (e.g., number of predictors) and complexity of the model against the unknown true sparsity and complexity in the data-generating mechanism (*13*), thus potentially allowing the application of ML methods to so-called fat datasets (when the number of features is much larger than the sample size) if the underlying signal is believed to be very sparse. Similar to non-ML analyses, careful processing of data (e.g., during the harmonization process), a deep understanding of where the data are coming from and what they can tell us (and what they cannot), is vital. The ML workflow has been described in several introductory papers; see (*1*) for a recent overview. Interesting in the light of legal requirements for data protection is the concept of federated learning, that is, decentralized training of ML algorithms without exchanging data across platforms. Federated learning needs a unified framework of harmonized data collection and analysis to be successful.

#### Feature selection

Feature selection is the usually researcher-guided choice of variables to be processed. This could be done based on domain knowledge, or data-driven by applying the minimal redundancy maximal relevance criterion or other feature selection criteria (*14*). Researcher-guided feature selection may be helpful in large datasets such as the Organisation for Economic Co-operation and Development’s multicountry and repeated cross-sectional Programme for International Students Assessment or longitudinal harmonized aging surveys from the family of health and retirement studies.

When selecting features for inclusion in the model, researchers should always be conscious of the curse of dimensionality, which describes the tendency of the test error to increase as the dimensionality of the problem increases, unless additional features are truly associated with the response (i.e., not just adding noise). More features, that is, variables in the model, increase the dimensionality of the problem, exacerbating the risk of overfitting. Thus, advances in data acquisition that allow for the collection of thousands or even millions of features are a double-edged sword; improved prediction can result if the features are truly relevant and the sample is population representative, but they will lead to more biased results if not. Moreover, even if they are relevant, the reduction in bias may be outweighed by increased variance incurred by their fit (*5*). The Donoho-Tanner phase transition of sparse recovery defines a sharp boundary of the sparsity/undersampling trade-off curve (*13*); in particular, there is an abrupt breakdown in model selection and fitting when the complexity of the model increases beyond a threshold, depending on the size and true complexity of the data. For the case of variable or feature selection, this implies hard limits on the degree to which the data analysis can be successful. Data-driven feature selection is characteristic for penalized approaches, which select features on the basis of their predictive ability and thus limit the number of features in the model.

#### Feature engineering

Conceptually interesting features, for example, cumulative risk (multiplicative effect of two predictors) or changes between measurements (e.g., weight loss over time), are not well detectable by simply adding them to the pool of variables. Features could be engineered, for instance, building difference measures or squared terms, on the basis of domain knowledge. A method for the investigation of systematic feature interaction, namely, the tree-based Random Forest feature importance and feature interaction network analysis framework, has recently been proposed (*15*). We additionally suggest to explore whether the reduction of complexity in the set of (related) independent variables makes sense, e.g., factor analysis or cluster analysis and/or selection of variables based on theory, to improve the ratio of features compared to units, and sample size suggestions have been proposed for specific research fields and methodological specifications (*16*). If the dataset is large enough, then a rule of thumb from our experience is the availability of several 10,000 relevant units (e.g., respondents to a survey); this enables the use of neural networks, which are well known to use feature engineering in the generation of the models.

While it is necessary to make the continuous variables equivalent in variance, we would recommend using manual feature engineering only to an extent to which researchers in the social and health sciences can still ensure some interpretability for real-world applications, particularly for the purpose of causal inference when exposures need to be well defined for their subsequent use in real-world interventions (*17*). Assuming that we have repeated assessments of body mass index (BMI) to predict or explain health, the model flagging BMI at assessment no. *x* (or *x* years before onset of disease) may not be meaningful in practice; however, after manually feature-engineering the slopes of BMI, the model flagging increases or decreases in BMI as predictive of disease may be very useful to identify at-risk patients. On the other hand, traditional modeling is highly depending on researcher decisions (e.g., modeling a quadratic instead of a linear relationship and modeling interaction effects manually). Here, algorithm-based decisions regarding feature engineering, for example, in neural networks, can provide more robust and accurate findings.

### Model building

In the process of model building, ML approaches will usually involve the three steps of (i) training, (ii) validation, and (iii) testing. Most researchers will be familiar with the ML modeling process, which comprises first splitting the data into a training and an independent test set and subsequently further splitting the training dataset into training and validation sets.

1) Training: The model parameters are estimated in the so-called training dataset.

2) Validation: Several trained models are assessed in a validation dataset to select among them the model closest to relevant metrics (e.g., complexity) and tune its hyperparameters.

3) Testing: The model is tested in a separate (held-out) test dataset to assess its generalization error. This measure gives an indication of how well the model will perform in future datasets on relevant performance metrics, for instance, accuracy of classification.

To improve both results from the validation and testing phases, cross-validation can be performed, a procedure used as well in more traditional statistical approaches.

**Fig. 1.**
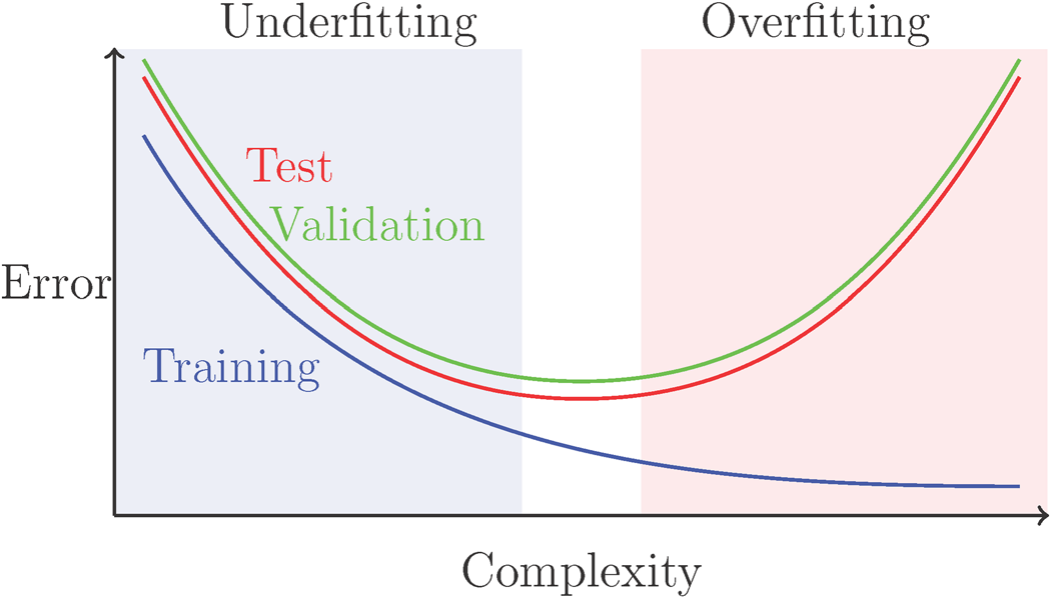
Typical relationship between model error and complexity. Copyright by Sara Wade.

Researchers need to be aware of the trade-off between increasing predictive accuracy and overfitting. The training error typically decreases with model complexity because of overfitting, while the test error curve has a U shape, decreasing at first because of underfitting and then increasing because of overfitting (Fig. 1). Although this might suggest choosing the model with minimal test error, it is important to never use the test data for model fitting or selection, otherwise this would give an unrealistic optimistic measure of performance. Thus, in the validation phase, the training data are further split into validation sets, and model complexity and tuning parameters are selected by minimizing the validation error. To improve estimation and to reduce variability, cross-validation, which averages over K-fold splits of the data, is standard in the validation phase. However, we note that cross-validation may also be performed in testing to improve estimation of the test error (*18*), especially when the sample size is small. To ensure that one never trains on the test data, this may require nested K-fold operations, using K-fold validation to choose the model within each test fold. Moreover, if cross-validated test errors are used, then the random splits should be reported for reproducibility and to allow other researchers to compare and test the methods. We illustrate the trade-off between predictive accuracy and overfitting in Fig. 1, which highlights the typical interrelation of training, validation, and test errors. We almost always expect the training curve to lie below the test curve, as most methods aim to minimize the training error. The validation curve typically lies above the test curve, because it is trained on a smaller training set, and can also be highly variable because of the random data splits, although cross-validation helps to reduce this variability. However, the goal of validation is to identify the correct level of flexibility, i.e., the minimum point of the test error. We note that cross-validation is appropriate under the assumption of independent and identically distributed data; for some data, such as time series or longitudinal data, this is not appropriate, and splits must account for the structure in the data. When the validation curve is relatively flat, the simpler model is preferred.

In the model building phase, many predictive ML methods will assign importance to features (variables) that increase performance more substantively than others. Researchers in the social and health sciences need to be aware that some ML methods, such as regression trees, will overvalue continuous predictors simply because of the availability of a larger number of possible splitting points. While rules for splitting decisions for categorical variables exist in some algorithms, they are handled differently across software packages. In contrast, approaches such as BART (described in more detail below) are supposed to handle the simultaneous inclusion of continuous, dichotomous, and categorical predictors. Experience suggests that continuous variables may however still be favored.

Model building can be done through user-friendly interfaces, which have been developed over the past decade. AutoML approaches aim to facilitate the ML workflow for non–data science researchers (*19*). AutoML, by default, trains and cross-validates generalized linear models, gradient-boosting machines, Random Forests, and deep neural networks and combines them via SuperLearning/stacking to improve the model fit. The Python software library scikit-learn lets researchers choose ML algorithms through a user-friendly platform (*20*). While easy-to-use interfaces are attractive to non–data science researchers and can be helpful in many cases, we argue that, similar to research with traditional inferential statistics, an understanding of the applied methods in greater detail is still necessary for substantial research contributions.

### Considerations for real-world applications of ML

Models developed on the basis of ML will, similar to models developed with more “traditional” modeling, inform prevention, diagnosis, treatment and care, and policy and practice in the social and health sciences. Stakeholders, users, and society at large need to accept ML for these real-world applications of ML—in one word, ML [and often called artificial intelligence (AI) in this context] needs to be “trustworthy.” Particularly relevant when setting up larger frameworks for ML-driven health care or social applications, ML and AI applications need to be lawful, ethical, and robust from both a technical and social perspective (*21*). We refer readers to discussions of “trustworthy AI” in recent publications (*21*). In the following, we will refer to three relevant concepts of trustworthy AI when applying ML in the social and health sciences, specifically (i) interpretability, (ii) fairness, (iii) generalizability, and (iv) ML to support human abilities and skills.

#### Interpretability/explainability and visualization

Interpretable ML means to extract relevant knowledge from ML models, i.e., able to feed into the domain knowledge of a discipline, characterized by predictive accuracy, descriptive accuracy, and relevancy, with relevancy judged relative to a human audience (*9*). Interpretability is particularly important in high-stake decisions, such as clinical decision-making. Instead of developing interpretable models separate from the original “black box” models, it has been suggested to only use interpretable models by design, as transparency and trustworthiness in the eyes of users can only be achieved with interpretable models (*22*). Interpretable AI is in more recent projects often called explainable AI or XAI (*23*).

Interpretability is, in practice, often linked with the possibility to visualize the estimated or found relationships among variables. To increase interpretability, bivariate and higher-order data associations with possible nonlinear patterns or missing data that are not at random and predictions can be visualized, e.g., with partial dependence plots (*24*), SHAP (SHapley Additive exPlanations) plots (*25*, *26*), or individual conditional expectation plots, a more recent and less well-known adaptation of partial dependence plots (*27*).

#### Fairness

A relevant notion in the health and social sciences is the idea of the ML algorithm to decide “fairly,” i.e., will not discriminate against certain social or minority groups. We refer to other literature for examples related to fairness in ML at large (*28*) and a prominent example of racial bias in a health care algorithm in use (*29*). Here, particularly in the application of ML in high-stake decision-making such as predicting recidivism, predictive model accuracy needs to be balanced against the model treating all social groups equally, i.e., the ML model resulting in all social groups having equal probability of receiving a desirable outcome. Importantly, health equity efforts can be undermined by structural discrimination/racism unintentionally implemented in ML-based decision-making. This concept of “algorithmic fairness” has received criticism. New proposals addressing the problems of producing fairness “within the data” suggest to define counterfactual fairness (*30*). A recent systematic assessment suggests that using fairness criteria harms model performance and at the same time leads to debatable improvements in fairness (*31*). The researchers thus recommend to consider the wider sociotechnical context that is possibly violating fairness outside the algorithms (*31*). Outside the scope of our review, it is critical to realize that fairness is not only important in application of algorithms but also in the full arc of the research process. This has been framed as an ethical pipeline from “fair” research questions, fair data collection, and fair data analysis to postdeployment auditing of ML algorithms to validate the “fairness” of the decision-making algorithms (*8*). Data inequality, i.e., smaller or lower-quality samples from members of minority groups in the field of biomedical health care research, has been addressed by training of the model on data of the majority group and then using of (knowledge) transfer learning to fine-tune model performance in the data of the minority group (*11*). However, some problems persist unless data inequality in health care data is systematically improved (*11*).

#### Generalizability or external validity

The importance of generalizability of external validity is not limited to ML approaches: Across research designs and across stages of data collection, we need to consider biases that could harm generalizability, as it is desired that findings have validity beyond the dataset in which they were found. In the context of ML, it is important to mention the risk of overfitting, i.e., to improve model accuracy within the dataset by risking model performance in a previously unused dataset. An example of how differences in recruitment in cohort studies can result in differential performance of ML algorithms is (*32*). In addition, similar to more traditional analyses, triangulation of methods is recommended. We further stress the importance of documenting all decisions in the statistical analysis to the details (e.g., specifying seed of random number) to ensure that scientific findings are replicable.

A particular case of generalizability is the assumption that the measured concepts, that is, our exposures or independent variables and our outcome of interest or dependent variable, are stationary, that is, not varying over time or depending on the input the model receives. In many settings, however, it is more likely that we need to be aware of possible concept drift, that is, a change in the input-output relation due to external factors. This is relevant in ML engines that continuously receive newly incoming data to process, which could be set up with health care, education, or consumer data (although admittedly not widely available in social and health sciences research at the time of writing). An example of concept drift over time would be the lowering of predictive power of poverty to explain children’s health when the societal determinants of poverty change over time; see an accessible introduction in (*33*) and recent developments in (*34*). While most research in the social and health sciences deals with static datasets to date, the importance of this concept will increase with the development of ML engines that continuously process newly incoming data, such as through tracking apps or social network data.

#### ML to improve human abilities and skills

There are numerous ML use cases to support humans at work or in their daily lives. Recommender systems are applied in multiple fields to suggest relevant items to users based on earlier information such as ratings of the same or other users. From the use of recommender systems across tourism, commerce, and marketing, we just present examples of how recommender systems can help work and health care: Recommender systems were used to overcome writers’ block in prolonged support chats of counselors with help seekers on suicide prevention hotlines; the setup seems promising to support emotionally strenuous work even if, at this point in time, human experts’ performance still exceeded that of the recommender system (*35*). We now move to the classification of ML approaches for description, prediction, and causal inference (some of which admittedly have their roots in standard statistical methods) and start with the description of ML approaches for description.

## ML FOR DESCRIPTION

A descriptive research question aims to “provide a quantitative summary of certain features of the world” (*6*). Description is the basis of all applied research, as we need description to quantify the phenomenon under study, e.g., assess prevalence or distributions of variables between (social) groups, countries, or geographical entities, or over time (between cohorts). This can be done through several algorithms (Table 1). Before moving to research goals in the social and health sciences with regard to description, we first introduce two relevant classes of methods, factor and cluster analysis, of which many researchers in the social and health sciences will already be aware.

**Table 1.** Overview and nontechnical description of ML methods for description most relevant in the social and health sciences.

**ML for description**
Clustering: Clustering identifies similarity structures in data and groups similar objects to previously unknown clusters, on the basis of specified criteria related to distance, density, and strategy of agglomerating smaller groups or partitioning larger groups. Examples are as follows: 1) k-means: Clusters data points according to a distance metric in an *n*(variables) dimensional space. 2) Hierarchical agglomerative clustering: Starting from each object forming a separate cluster, clusters are consecutively merged moving up the hierarchy. 3) Model-based clustering: The most widely used example is the Gaussian mixture model (GMM), which generalizes k-means by allowing an elliptically shaped cluster. 4) Density-based spatial clustering of applications with noise (DBSCAN) (*131*, *132*) groups data points in spatial proximity while marking data points in low-density regions as outliers. 5) Mixtures of experts (learners): This approach divides the input space in homogeneous regions, and a different expert is responsible for each region. This allows for different clusters, e.g., of patients, with different (non)linear relationships between *y* and *x* (*133*, *134*). Dimensionality reduction 1) Principal components analysis (PCA): Known to researchers in the social and health sciences, this method provides a low-dimensional approximation/encoding of the data by linear (orthogonal) projection (i.e., low-dimensional features are linear combinations of the original features). 2) Probabilistic PCA (PPCA): Probabilistic model formulation of PCA for higher-dimensional data such as found in metabolomics. The PPCA will provide the PCA solution in the limit of zero noise (*135*). 3) Factor analysis: Also known to researchers in the social and health sciences in the analysis of, for instance, questionnaire-based data, factor analysis can be seen as a generalization of PPCA that allows dimension-specific noise. This method explains the correlation across dimensions through a small number of latent factors. 4) Independent component analysis: Generalization of factor analysis that allows the distribution of the latent factors to be any non-Gaussian distribution. 5) Nonlinear dimension reduction: Includes kernel PCA, Gaussian process latent variable model (GPLVM), and t-distributed stochastic neighbor embedding (*t*-SNE) (*36*) 6) Generative adversarial networks (GANs): The task of grouping data points on the basis of similarity is split into a two-part problem of, first, generation of new data that should be similar to the real data and the task of, similar to supervised learning, classifying the data as either real or new (fake). The task stops once the algorithm is no longer able to discriminate real from new data (*136*). 7) Variational autoencoders (VAEs): Use neural networks for dimensionality reduction, both for encoding and decoding (mapping the data to the low-dimensional latent space and vice versa). VAEs use a probabilistic formulation and variational inference to learn the distribution of the latent variables, which avoid overfitting and impose desirable properties on the latent space (*137*). Anomaly detection This is the process of identifying data points that deviate from normal “behavior,” that is, is identified as dissimilar in the context of the overall data points. Anomalous data may indicate an incident, deviant behavior (e.g., fraud in data of bank transfers and changes in household composition of a consumer in consumption data). Anomaly detection can be a specific form of social network analysis (SNA) (see below). Biclustering As a more recent development, one may also be interested in the simultaneous grouping samples (individuals) and features (variables) based on similarity. The so-called biclustering methods simultaneously cluster samples and features; for a recent review, see (*138*). Biclustering is used in bioinformatics, e.g., to cluster patients on the basis of expression profiles on a subset of genes (*139*). Social network analysis SNA assesses the connections or relationships (edges) between different data points (nodes; e.g., users, voters, co-workers, and organizations). SNA describes networks with structural or content-based measures; see (*53*) for an overview. Examples of structural measures are centrality, which assesses the relevance or structural importance of a node in the network and is captured through, e.g., degree centrality (nodes with more connections are ranked higher) or eigenvector centrality (adjusts for importance of neighbors), or group centrality, which generalizes the centrality measure to a group of nodes; again different assessments are possible, such as group degree centrality. Content-based analysis may extract user profiles or conversation topics.

Dimensionality reduction can reduce complexity of datasets for more efficient subsequent analysis (*36*). Factor analysis will provide factors and factor loading for the included variables. Factors are often used as variables with densified information in subsequent analyses. Readers should note that algorithms based on Bayesian modeling are available for factor analysis and other methods presented here but will not be covered in detail. Domain knowledge is necessary to preselect variables for factor analysis that are interpretable and meaningful, as cluster (and factor) analysis cannot conceptually distinguish variables, for example, if data are coming from humans or from other sources. We suggest to not mix individual-level variables with higher-order variables, e.g., related to environment or neighborhood in a factor analysis to ensure interpretability of the factors in subsequent analyses. Another helpful approach may be cluster analysis to group data on the basis of similarity. Unlike in factor analyses, cluster analysis can help to process measurements of individual-level and contextual-level variables simultaneously, for example, individual-level BMI and contextual-level air pollution to investigate different risk groups or profiles (e.g., with high BMI and high air pollution) for cardiovascular mortality. Last, biclustering may be helpful if samples and variables need simultaneous grouping.

Descriptive research goals can be found in the social and health sciences in (i) the screening and identification of at-risk individuals or higher-level patterns, (ii) the identification of risk profiles, (iii) the estimation and projection of prevalence of adverse outcomes, and (iv) the research goal of diagnosis. These four research goals will be described in the remainder of the section.

### Screening and identification of at-risk individuals or higher-level patterns

Individuals can be screened for single risk factors, through automated processing of data; for example, people at elevated risk for adverse health outcomes can be identified through processing of electronic health records. This research goal could be solved with algorithms for anomaly detection. Conducting clustering or factor analysis, a group of variables can be processed simultaneously, by analyzing patterns across individuals. To identify aging-related morbidity pathways, electronic health records that contained information on 278 high-burden diseases were analyzed with different clustering algorithms to group diseases according to their patterns of age at onset of disease (*37*). To add, the complex information derived from ML was visualized with traditional plotting, for example, distributions of onset of disease curves per disease cluster (*37*).

Longitudinal analyses of trajectories of time-varying variables can be helpful to better understand the trajectory of previously identified risk factors with short-term or long-term observational data, preferably with a minimum of five follow-up measurements. Many social and health phenomena undergo changes over the life course, the trajectories of which can be considered normal development (e.g., increases in cognitive skills across childhood and adolescence and decreases thereof in later old age). Other changes, however, may be predictive of disease (e.g., strong weight gain, strong weight loss, or cognitive decline in midlife) and thus be relevant in interventions and practice. Short-term repeated assessments may be relevant in situations where continuous monitoring and intervention may be necessary, for instance, in the surgical context (*38*), and trajectories of monitored factors may bring additional insights into the status of patients. Long-term repeated assessments can be interesting in diseases where long-term risk prediction is relevant or where long prodromal phases condition the need to distinguish whether factors are indeed risk factors or early symptoms. Depressive trajectories were identified via k-means clustering of the number of depressive symptoms at each measurement occasion over a long follow-up to distinguish early-onset from late-onset depression and to test their associations with adverse brain outcomes (*39*). Another study used fuzzy clustering of data of patients with first-episode psychosis to identify four trajectories “excellent prognosis,” “remitting course,” “clinical worsening,” and “chronic course,” with distinct risk factors for worsening and remitting course (*40*). Analyses similar to this can help elucidate changing risk factor importance and their interactions over the life course.

Death as a competing risk needs to be accounted for in investigations on aging-associated diseases, e.g., with random survival forests, which also allow modeling of time-varying risk factors (*41*). If a focus is on the short-term consequences of time-varying treatment, i.e., if causal conclusions are intended, then it may be better to use established methods in the potential outcomes framework such as marginal structural models or the g-formula (*42*).

### Identification of risk profiles

To identify risk profiles, i.e., groups of individuals characterized by certain values on a set of variables, ML for discovery can be used to describe and reduce complexity, based on previous literature that had identified risk or protective factors. Using severity scores common in the intensive care unit setting, patient health state trajectories were categorized with a number of dimensionality reduction (and predictive) techniques in time series data, among others, density-based spatial clustering of applications with noise (DBSCAN) (*43*). These health state trajectories were correlated with medication and treatments, with commendable visualizations of the resulting patterns (*43*).

While most descriptive research problems in the social and health sciences will require unsupervised ML, there is no one-to-one correspondence of descriptive problems to unsupervised ML. In the following, we will present descriptive research questions that need supervised ML approaches typically used for prediction.

### Estimation and projection of prevalence of social or health outcomes

Estimating disease prevalence is the basis for quantifying health burden, the need for interventions, and health and social care planning. Estimation of the rate, incidence, or prevalence of a phenomenon under study, for example, a health outcome such as diabetes in a certain population, can be considered descriptive; the projection of estimates would rather be predictive. An example, incorporating the social determinants of health perspective, is to take prevalence estimations of noncommunicable diseases (NCDs) by age, sex, and race/ethnicity (can be done with individual-level data or aggregate data) and estimate their prevalence for unknown areas through ML. The presence of six NCDs was estimated through LASSO to predict population-level prevalence of NCDs with a minimal demographic dataset for 50 U.S. states (*44*). Conversely, Wang and Rodriguez (*45*) used log-linear models with a generalized fussed LASSO penalty on the spatial random effects to identify spatial disease clusters, i.e., regions of higher occurrence of greater than the expected number of cases of a disease, in this case, pediatric cancer, in Florida. Another study used recurrent neural networks, more specifically long short-term memory and gated recurrent unit networks for disease activity monitoring, in this case, influenza disease outbreaks, with multiple spatial resolutions across the United States (*46*).

### Diagnosis

A descriptive research goal is to identify prevalence of a social or health outcome. A diagnosis related to a health condition can often be reasonably inferred also in the absence of clinical assessment on the basis of indicators that would be a relevant marker for the disease (e.g., high blood sugar for diabetes). An inferred diagnosis can be termed to be “probable” on the basis of the available information. In the following, we will present the goals of (i) assessment of probable diagnosis, (ii) addressing underreporting or underdiagnosis, and (iii) identification and spread of social or cultural influences within a network.

#### Assessment of probable diagnosis

The descriptive goal of identifying individuals (respondents) with a probable diagnosis of a disease is a relevant goal in data-scarce environments due to lack of resources or data privacy issues. The goal can be accomplished with supervised ML or a semisupervised setting if a mix of labeled and unlabeled data is analyzed. With data from electronic health records, algorithms can, in the absence of human clinical assessment, identify the existence of characteristics (or joint presence of conditions) that increase the likelihood of a presence of disease, for example, through neural networks. Other data, such as sensor data and language data, can be used to detect conditions or disease, through methods not covered in this review, such as natural language processing, which has been, among many other applications, used to detect depression in social network data (*47*).

In the absence of a diagnosis based on clinical assessments, classifying individuals with a probable diagnosis through ML may be interesting to estimate population-level disease prevalence and associated health care costs. Identifying individuals with probable diagnosis is unobtrusive and may be more cost-effective than the clinical assessment of these individuals. Combining principal components analysis and cluster analysis, participants with a high likelihood of dementia were identified with datasets from across the world (*48*). With cultural and education fair battery of cognitive tests, the 10/66 diagnosis of dementia can be done with cognitive tests in the absence of possibilities for clinical assessment, with application of ML to data from South India (*49*).

#### Addressing underreporting or underdiagnosis

Supplementing classifications of probable diagnosis with diagnosed individuals may address underreporting and underdiagnosis of conditions such as dementia. These individual-level probable diagnoses can be used to investigate risk and protective factors of these conditions. Survey participants were identified as having probable dementia with a mix of traditional and ML-based (descriptive and predictive) algorithms and with the aim to provide dementia classification algorithms with similar sensitivity/specificity across racial/ethnic groups (*50*). ML in this study proved more complex to implement and was considered more sensitive to cohort and study procedural differences than traditional expert modeling, that is, feature selection based on domain knowledge; in addition, the use of ML, in this case, LASSO and the SuperLearner, did not lead to increases in model performance compared to different expert models (*50*). Samples of less than 2000 participants with a long time frame may thus be less recommended for the use of ML approaches.

### Identification and spread of social or cultural influences within a network

A particular research question related to diagnosis may be the identification and spread of social or cultural influences within a network, which can be solved with social network analysis (SNA). Among multiple empirical studies, we can give only a few selective examples of how SNA has been a helpful tool with classic statistical analyses in the social and health sciences to analyze the determinants of spread of political attitudes in social networks and beliefs and collaboration in and between organizations.

In the framework of SNA, specific ML methods have been developed such as exponential family random graph models (*51*), but we find many examples of SNA being carried out with ML methods developed for prediction, such as support-vector machines or deep learning (see Table 2). SNA has been used to measure political orientation and identify user profiles and conversation topics through content-based analysis; a nice example of exploiting both approaches to understand (less than expected) segregation on social media by political orientation is the study of Barberá *et al*. (*52*). An overview of SNA together with a categorization into four different dimensions of SNA evaluation, namely, pattern and knowledge discovery, information fusion and integration, scalability, and visualization, can be found in (*53*). ML methods applied in the SNA context may be evaluated with regard to their performance similar to their application in predictive contexts, for example, regarding accuracy or precision (Table 4). In the following section, we will describe ML approaches for the purpose of prediction.

**Table 2.** Overview and nontechnical description of ML methods for prediction most relevant in the social and health sciences.

**ML for prediction**
Regressions: 1) Linear regression: Predicts continuous outcome by adding an intercept to a weighted sum of predictors and their estimates, fitted most commonly with ordinary least squares, minimizing squared prediction errors. 2) Logistic regression: Predicts categorical outcome probability; uses logit function as link in a generalized linear model; fitted most commonly with maximum likelihood or gradient descent; and can be extended to reflect more than two outcome categories. Artificial neural networks and deep learning: Artificial neural networks search for complex patterns in data with a large number of data points (variables and individuals) to build connected units or nodes, mimicking a simplified brain structure of interconnected neurons. 1) Convolutional neural networks: Apply filters to smaller units of grid-structured data (e.g., imaging or sequential data) and translate the corresponding activation into a feature map; computationally expensive and data intensive; outperform approaches to imaging analysis that require manual feature crafting or image segmentation. 2) Multilayer perceptron: Most prominent artificial neural network consisting of neurons organized in one input layer, at least one hidden layer, and one output layer; neurons of one layer contain weighted summaries of neurons in the previous layer, calculated with an activation function; backpropagation describes the process of how weights are adjusted to minimize the observed error in the output layer. Ensemble methods: 1) Random Forest: Apply bootstrapping concept to individual decision tree, based on random subsets of the input variables; prediction is based on majority votes in the resulting forest of individual decision trees. 2) XGBoost: Software- and hardware-optimized implementation of gradient boosting machines that minimizes prediction error of a sequential ensemble of individual decision trees, outperforms Random Forests, and is especially well suited for structured, tabular data. 3) Bayesian Additive Regression Trees (BART): Embeds a sequential ensemble of individual decision trees in the Bayesian framework, allowing to assess uncertainty of estimates; sets statistically robust defaults and thus does not require manual regularization or hyperparameter tuning; and can also be used for causal inference. 4) SuperLearner: Uses multiple descriptive and predictive ML algorithms and estimates the respective performance through cross validation; the accuracy of SuperLearner approaches the best prediction algorithm tested (*61*). Support-vector machines: Support-vector machines (SVMs) have been among the most widely used supervised learning methods for classification or regression (*140*). SVMs train a model by finding hyperplanes between data points (vectors) and choose the hyperplane that most clearly separates data points of one category from the other (in other words, with the largest margin), i.e., the distance to data points of each category is maximized. SVMs remain robust prediction methods even if outperformed in recent years by gradient boosting and other ensemble algorithms (*141*). Regularization algorithms: 1) Ridge regression: Adds a regularization term based on the regression coefficients to the sum of likelihoods (maximum likelihood) or sum of squared residuals (ordinary least squares) during model fitting; thus introduces bias to reduce variance in large models, i.e., shrinks parameters; can be applied, e.g., to linear or logistic regressions; and performs well when most variables are informative. 2) LASSO: Similar to ridge regression; useful for variable selection because it can shrink estimates of noninformative variables to 0, whereas ridge regression can only shrink estimates asymptotically toward 0; performs well when many variables are noninformative. 3) Elastic net: Introduces regularization based on both LASSO and ridge regression; performs well when variables intercorrelate and informativeness of variables is unknown. Decision trees: 1) Classification and regression tree (CART): Individual decision tree; data are iteratively split into nonoverlapping bins according to split or cut points; these refer to predictor values determined with a greedy algorithm; prediction is based on mean values or majority votes in terminal/leaf nodes reached by traversing the respective path in the tree; base element of, e.g., Random Forests. 2) Conditional inference tree: Similar to CART; variable selection for recursive partitioning is based on significance test, whereas CART selects split variables based on maximization of information measures such as the Gini coefficient, which is a measure of statistical dispersion often used in research on income inequality.

## ML FOR PREDICTION

Prediction problems are highly relevant in social and health sciences: We may want to predict a certain output, that is, social or health outcome, either as accurately or as parsimoniously as possible. Research goals may be to explain the maximum variance in the outcome or find a minimal or optimal predictor set to improve the identification of at-risk individuals. We may want to evaluate how well a certain input, for example, a candidate risk factor, is able to predict an outcome. Prognosis in its simplest form is a prediction problem. There may be defined end points, and we wish to estimate the probability of reaching one of the end points. With a perspective of ML as letting the computer/the algorithm define the model instead of the human, ML can test the relative importance of one or more predictors, by considering a large set of covariates, and provide absolute values of importance or rank order information. Again, the curse of dimensionality mentioned above applies. For in-depth explanations, we refer to prediction textbooks (*12*).

**Fig. 2.**
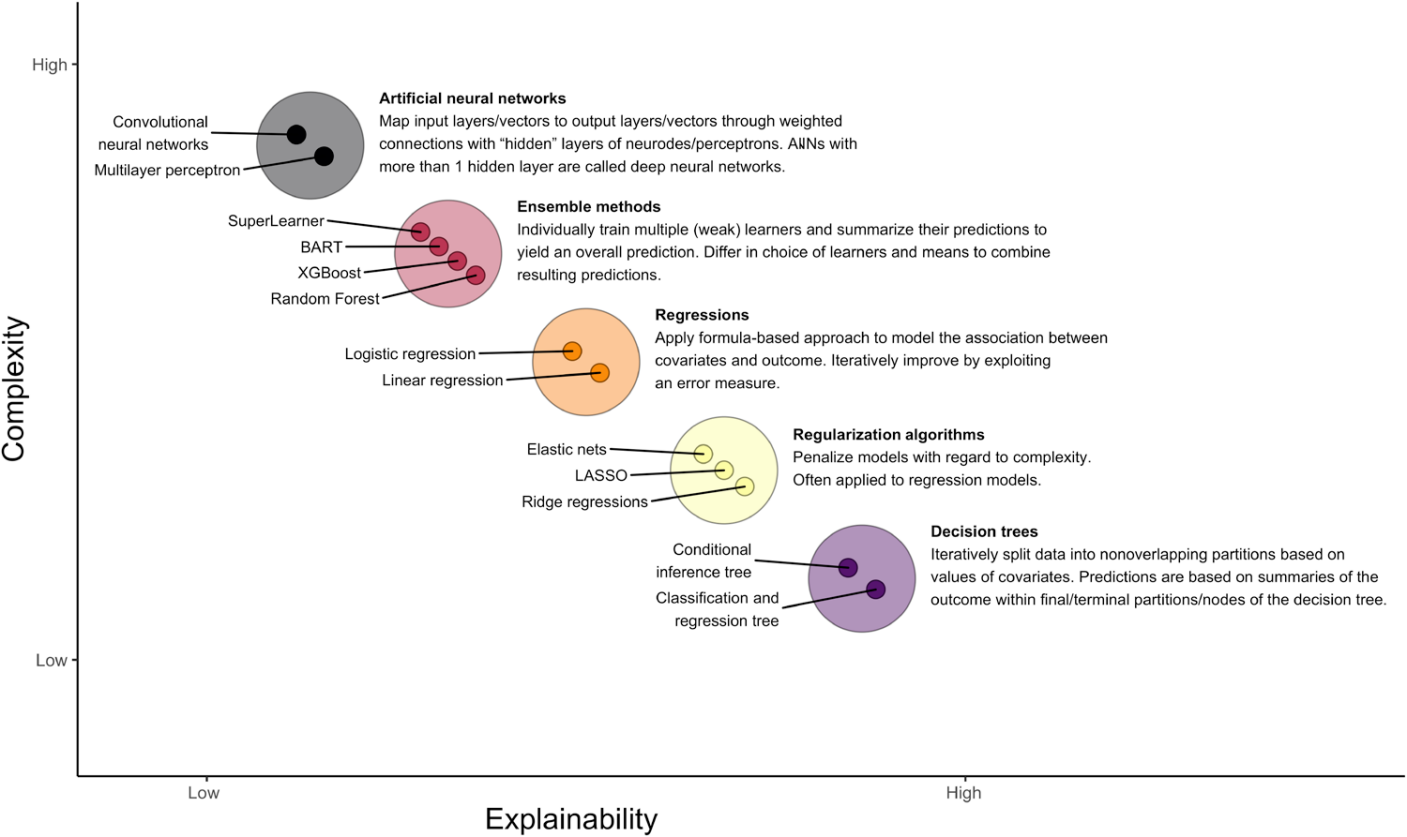
ML methods for prediction most relevant in the social and health sciences with nontechnical description ranked by interpretability/explainability versus complexity. Note that classes of methods are represented as larger circles; specific ML methods are represented as small circles within. Ordering and selection of ML methods based on theoretical considerations and experience. ANN, artificial neural network. Copyright by Matthias Klee.

The research purpose of prediction will need mapping some features (input) to other, known, features (output) as accurately as possible (*6*). Again, known outcomes such as a health outcome are called labeled data; to investigate predictive research questions, we thus use supervised learning: As explained above, supervised learning is the ML task of learning a function from labeled data to map an “input” (predictors and independent variables) to an “output” (outcome). Numerical (continuous) outcomes will require regression techniques, while dichotomous or categorical outcomes will require classification techniques. Figure 2 presents an overview of ML methods that are, on the basis of theoretical considerations and experience, ranked to explain the trade-off between explainability versus complexity of these methods. Before presenting research questions in the social and health sciences specific to prediction, we give an overview of common ML approaches typically mentioned for prediction.

### Common predictive ML approaches

As a few popular and/or well-performing ML methods, typical ML methods to solve predictive problems are (i) penalized approaches, (ii) ensemble learning, and (iii) neural networks, which are described in more technical detail below. More approaches are presented in Table 2.

#### Penalized approaches

Shrinkage or penalized regression methods are also used in traditional inferential statistics. Penalized regression performs better than the standard linear model in large multivariate datasets where more variables than individuals are available. Penalized regression will add a constraint in the equation to penalize linear regression models with too many variables in the model, also called “shrinkage” or “regularization.” This will shrink the coefficient values toward zero, so variables contributing less will have coefficients close to zero or equal to zero (*4*). The most widely used penalization methods, corresponding to different shrinkage penalties, include LASSO, ridge regression, and elastic net. However, while LASSO thresholds some coefficients to zero, thus simultaneously performing variable selection and estimation, it suffers from a well-known estimation bias. To overcome this, other penalties have been developed, including smoothly clipped absolute deviation (*54*). In addition, various extensions have been proposed to account for structure in the data, such as fussed LASSO for temporally structured variables (or more generally graph-structured variables) and group LASSO for group-structured variables.

#### Ensemble learning

Common ensemble learning algorithms are Random Forests, XGBoost, and the SuperLearner, which will be presented in the following. BART is also an ensemble learning algorithm; however, they are often used for causal research questions as relevant software packages offer numerous settings for causal analysis based on domain knowledge (*55*, *56*) and will be presented in the “ML for causal inference” section. First, because of their interpretability, ensemble methods such as Random Forests (*57*), have been used in numerous studies in the social and health sciences such as (*41*, *58*).

Second, commended for its high predictive accuracy and robust applicability to many predictive problems, we point readers to stochastic gradient boosting (*59*), which has been implemented in ready-to-use packages in Python and R for the family of gradient-boosting algorithms, for example, in the recommended “xgboost” package. Through boosting, the typical problem of collinearity of input variables (predictors) does not occur, which means that differently engineered (preprocessed) variables can be entered simultaneously to see which characteristics are most predictive. Researchers need to be aware that the algorithm does an exhaustive search over all variables for splitting points, and some variables may be more informative to divide the sample than others. The algorithm is thus biased toward choosing numerical (continuous), multicategory variables or variables with missing data over dichotomous variables; methods toward unbiased variable selection are available (*60*). Furthermore, variables will be picked as splitting points that best explain the dependent variable, which is not necessarily the most meaningful variable from a theoretical perspective relevant in the social and health sciences.

Last, some ensemble learning algorithms capitalize on the possibility to test the performance of models developed across multiple ML algorithms. An example family of algorithms is the SuperLearner that has been applied in epidemiological research questions (*61*). The SuperLearner uses cross-validation to estimate the performance of several descriptive and predictive ML models, or in the same model with different settings, and works asymptotically as accurately as the best prediction algorithm used in the model fitting process. One application to this is the imputation of missing data (*62*).

#### Artificial neural networks

Artificial neural networks typically use a large number of data points to search for complex patterns and build connected units or nodes. Artificial neural networks with more than one hidden layer are also called deep learning.

##### Detailed description: Artificial neural networks

A more complex way of solving prediction problems by mapping some features (predictors) to other features (outcome) can be done with neural networks, which learn interrelationships between variables, with a defined input (predictors) and “result” (outcome). Neural networks are motivated by the computation in the brain that enables successful recognition and classification of complicated tasks (*63*). Neural networks are identical to the traditional logistic regressions with no hidden layer if the logistic activation function is implemented, which is the most common case (*64*). Both neural networks and logistic regression have a functional form, and the parameter vector is determined by maximum likelihood estimation. However, neural networks allow us to relax the linearity of input variables and log odds assumption. Consequently, it is a better option if the data are not linearly separable. This flexibility comes with a cost of difficulty in interpretation of the parameters; the resulting model is evaluated through model performance measures such as sensitivity, specificity, accuracy, and the area under the receiver operating characteristic curve (see Table 4). Neural networks build at least one hidden layer between the input and output, and the benefits of neural networks to increase model performance actually come from the algorithms’ capacity to develop several hidden layers. The training process of neural networks mainly consists of two steps. First, feed forward takes the inputs or the previously hidden layer and combines them with weights. Second, backward propagation takes the output layer or its previous hidden layers to adjust on the basis of the error between the actual and the predicted values. By iteration of this feedforward and backward propagation, neural networks train to adapt the transformation and regression parameters. If no careful process of testing and cross-validation after training is implemented, then neural networks are susceptible to overfitting. Regularization can solve this problem through cross-validation or bootstrapping (*65*). Another way is to use the Bayesian framework. Rather than giving a point estimation, it calculates the distribution of parameters to avoid overfitting problems (*66*). Moreover, while neural networks tend to be overconfident even when predictions are incorrect and are vulnerable to adversarial attacks (*67*), Bayesian neural networks, which produce an ensemble of neural networks, are robust and accurate (*68*). This may be particularly relevant to increase trust and social acceptance in social and health sciences also in the light of the trade-off in interpretability due to the complexity of the algorithms and resulting models. In the following, we present research questions related to prediction and examples from the social and health sciences, specifically (i) prediction of social or health outcomes, (ii) identification and evaluation of risk factors, and (iii) identification of processes and deviations from “normal” processes.

### Prediction of social or health outcomes

Very often, the first step in understanding a social phenomenon or a disease will be to predict who is going to show the outcome, taking into account the presence of sociodemographic, psychological, health, or relevant other determinants (predictors). We first discuss research with the main interest in the (i) output, that is, research with the aim to predict a most often adverse, but depending on the research questions sometimes also beneficial, social or health outcome as accurately as possible. We then move to research questions to find (ii) minimal or optimal predictor sets and (iii) suggest improvements for the prediction of rare outcomes.

#### Accuracy of predicting adverse outcomes

Model building to predict adverse outcomes will usually involve training on a large, researcher-selected set of features. Possible improvements of model performance should be tested by building additional models, adding more information (variables) to the model, or by accounting for higher-order interactions or nonlinear relationships. Typical research questions have been to test improved predictive accuracy of ML compared to traditional modeling, e.g., regarding the social determinants of health (*58*), the prediction of dementia (*41*), and diabetes and diabetes complications (*69*), with ML approaches typically not markedly outperforming traditional regression-based modeling. Other studies have however shown that, for instance, estimation of remaining life expectancy with a long short-term memory recurrent neural network using electronic medical records outperformed human (doctor) estimations; remaining life expectancy is a relevant patient-oriented measure for timely start of advance care (end-of-life) planning (*70*). A good illustration of this research goal is a retrospective prediction of veteran suicides. BART was the best ML algorithm of several tested in a dataset of veteran suicides matched with a 1% random matched sample of veterans alive at the time (*71*). Other research has used the SuperLearner for mortality risk prediction (*72*). A systematic review on clinical risk prediction with ML found little benefits of ML methods over regressions and criticized a number of shortcomings in the literature up to that date, particularly the lack of calibration, i.e., testing the reliability of predictions (*73*). Researchers may be aware that the more distal the predictors, the more difficult it will be to arrive at robust, accurate predictions. As an example, a study used characteristics of correctional facilities and aggregate inmate characteristics to predict prison violence, assessed by the number of inmate-on-inmate assaults with the SuperLearner but failed to arrive at high levels of accuracy (*74*). Spatial mapping in social geography was shown to be possible with parsimonious data; for example, a study using mobile phone data mapped poverty and wealth geographically and arrived at distributions similar to those gained from boots-on-the-ground survey data collection (*75*).

In clinical practice, the relevant output may not be an adverse health outcome but the necessity and optimal timing to intervene. Prediction of optimal timing of clinical decisions has been researched, for example, by the van der Schaar laboratory, which has developed automated ML architecture, such as the so-called AutoPrognosis, to predict adverse cardiovascular outcomes better than traditional risk scores (*76*).

#### Minimal and optimal predictor sets of social or health outcomes

Interest in the output, that is, the social or health outcome, may also come with the aim of finding a minimal or optimal (most parsimonious) predictor set. Coming back to the earlier mentioned example to predict population-level prevalence of NCDs, these diseases were estimated with a minimal sociodemographic predictor set (*44*). In dementia risk prediction, for which, until recently, no robust algorithms were available, an optimal model predicting dementia over 10 years has been recently developed with LASSO (*77*). An optimal predictor set was sought in one study to explain variance in firearm violence, combining LASSO and Random Forest algorithms (*78*).

#### Prediction of rare outcomes

A typical problem in the health and social sciences is the prediction of rare outcomes, such as disease, crime, learning difficulties, and divorce, where only a very small percentage of the observed population will show the outcome of interest. For example, the rate of offenders is very small compared to the total population; autism is prevalent in around 2% of children; up to one-third of married people will file for divorce. Using ML to predict rare outcomes (and rare may be defined as anything less frequent than 50% of the cases), classification algorithms will usually simply develop a model that will only predict the nonoccurrences of the outcome, because the algorithm will detect that a guess of “0” will be correct in most cases. Researchers may solve this problem through several strategies:

1) Redefining the outcome, for example, as a regression instead of classification problem, for instance, using the full range of a depression scale as continuous or Poisson-distributed variable instead of the binary (and rare) classification of clinically relevant moderate/severe depression.

2) Changing the distribution of the outcome in the sample by oversampling of the group with the rare outcome through, for example, synthetic minority oversampling technique (*79*), is well established. Equally, downsampling (undersampling) of the group without the outcome is possible. An example of highly imbalanced data is financial services consumer behavior, where prediction of mortgage default and identification of high-risk consumers are crucial for banks and lending companies, respectively. A recent study undersampled nondefaulting customers in consumer transaction data and trained convolutional neural networks, which in combination with Random Forests led to the highest predictive accuracy (*80*). Some studies report repeated random subsampling of highly imbalanced data to predict diseases in a health care dataset, with Random Forests outperforming support-vector machines, bagging, and other approaches (*81*). It should be noted that, in the case of cross-validation, sampling strategies should be implemented within folds. On a more general note, sensitivity checks are required to investigate the impact of different sampling strategies on the performance and generalizability.

3) A rare outcome may be more frequent in preselected samples; for example, dementia research has partly focused on improving prediction of conversion from mild cognitive impairment (MCI) to dementia. Sample selection can only be successful if conclusions drawn from the analysis do not need to hold for the whole population, otherwise this strategy would introduce a bias (conditioning on the outcome). At-risk samples of people with MCI may be used to train a model that discriminates converters from nonconverters on the basis of questionnaire-, biomarker-, or imaging-based variables. Among numerous examples, some studies tested the role of cognitive reserve to predict conversion to dementia with different ML algorithms (*82*) and tested their model developed with a support-vector machine algorithm in previously unseen subjects (*83*). While dementia risk prediction models improve substantially particularly after adding genetic and imaging information to the models, there is no significant progress in the ability to predict decline of cognitive performance tests in at-risk samples (*84*).

4) Simulated datasets and “virtual cohorts” may be useful in some cases.

### Identification and evaluation of new or known risk factors

With an interest in the input, that is, predictors of a social or health outcome, some research has used large predictor sets to identify previously unknown predictors of a social or health outcome or to evaluate their predictive ability in the context of the other variables. Aside from the curse of dimensionality that needs to be considered, testing the previously untested predictor set with several ML approaches may be helpful to balance limitations. Studies with this aim have tested, for example, candidate modifiable factors associated with childhood cognitive performance (*85*). In another application, gradient boosting and SHAP plots were used to identify the top 10 risk factors of suicidal thoughts and behavior in adolescents, all related to sociodemographic factors and family and peer relationships (*86*).

A study investigated lifestyle factors known to be linked with cognitive functioning, measured by wearables, on their association with cognitive functioning assessed through Mini–Mental State Examination (MMSE) scores (*87*). While no causality could be established because of the cross-sectional nature of the study, partial dependence plot visualizations revealed nonlinearities, such as associations plateauing off after a certain threshold or associations with inverse-U relationships (*87*). Studies as this help to improve our thinking about expected and actual relationships in the exposure-outcome associations, i.e., are the benefits we expect from a certain lifestyle continuously increasing (linear or quadratic dose-response relationship) or leveling off after a certain value, after which no further improvements can be expected (threshold model)? Fine-grained assessments of both exposure and outcome are necessary for these investigations. A study used neighborhood characteristics to predict opioid overdose mortality with LASSO, finding previously unidentified relevant neighborhood characteristics, such as residential stability, racial/ethnic distribution, and social isolation (*88*). Another study tested the associations of childhood adverse experiences with intelligence in a cross-sectional design (*89*). Moving from the chosen term “risk factors” to the more neutral “determinants” or “predictors,” another study, aiming at identifying the drivers (predictors) in human decision-making, used a large-scale experiment on risky choice to test classical decision theories through deep (artificial) neural networks (*90*).

### Identification of processes and deviations from normal processes

Researchers may be interested in moving beyond descriptive research when investigating trajectories of social and health outcomes and instead adopt a predictive lens if different states or trajectories are already defined by the topic, for example, disease severity or educational or occupational level. A study investigated predictors of chronic obstructive pulmonary disease of highest severity and common disease trajectories with gradient boosting and a shifting time window approach in health claims data: The authors identified a number of diagnoses (e.g., respiratory failure), medications (e.g., anticholinergic drugs), and procedures associated with a subsequent chronic obstructive pulmonary disease diagnosis of highest severity (*91*). The temporal patterns detected in this study rather represent order of health care–relevant diseases and should not be interpreted in the sense of causal pathways (*91*). In other contexts, detected temporal patterns may be more robust.

Researchers with an interest in processes of aging may want to define normal aging-related trajectories of social or health outcomes. Then, deviations, defined as a predictive problem, from the normal trajectory could be identified, for example, with gradient boosting (*92*). Researchers should be aware that this goal poses strong requirements on data, as defining the normal aging trajectory is not trivial. Ideally, enough information is provided to ensure interpretability and replication. In the following, we will describe research in the social and health sciences, aiming with a causal lens.

## ML FOR CAUSAL INFERENCE

Much of what social and health science researchers are after is related to finding causes of a certain feature of the world or consequences of a certain feature of the world; so, often, these disciplines will seek answers to causal questions. We want to identify not only predictors but also risk or protective factors, for example, to use in prevention of an adverse social or health outcome. If we do not only want to understand determinants but also intervene, then we need an understanding of causal determinants of the disease.

Multiple requirements to the statistical analysis need to be fulfilled before satisfying answers can be found to the question of causality. ML for causal inference requires domain knowledge or, in other words, subject matter expertise. It is vital to select variables wisely according to their position on the directed acyclic graph (DAG) describing the assumed causal relationships between variables (*93*, *94*), which is more and more applied in health research (*95*). Any statistical analysis aiming at causal inference will usually select datasets in which assumptions of causal inference can be assumed to be fulfilled: exchangeability (ignorability), i.e., for all who did not receive a particular treatment, the outcome would be the same as for those who did receive the treatment had they been treated (counterfactual probability of outcome), positivity, i.e., all possible values of every level of exposures for every combination of values of exposures and confounders are available or have been assigned in the dataset; and consistency, i.e., an individual’s potential outcome under their observed exposure history is precisely their observed outcome (*96*). However, even in contexts of (limited) violations of these conditions, some research progress could be made by defining hypothetical interventions or “target trials” if randomized controlled trials are not an option from an ethical perspective or not feasible for other reasons. Using observational data with a potential outcomes framework, we can emulate a target trial (*97*, *98*). A recent study emulated a target trial to test the effects of interventions of modifiable factors on BMI rebound in childhood using targeted maximum likelihood estimation (TMLE) to estimate coefficients (*99*).

In line with the categorization of Hernán *et al.* (*6*), we first consider here ML for counterfactual prediction, but we will extend their framework by also considering ML for causal discovery, that is, methods, in which the causal structure between variables is learned from the data (Table 3). Using the framework of structural causal models has a large potential for applications in the social and health sciences, even if largely unexplored today.

**Table 3.** Overview and nontechnical description of ML methods for causal inference most relevant in the social and health sciences.

**ML for causal inference**
ML for counterfactual prediction Setting up data to meet assumptions of causal inference and use predictive ML (Table 2) for effect estimation. Several packages offer commands supporting the estimation of causal effects: 1) BART, see the detailed description above. 2) Targeted maximum likelihood estimation (TMLE): In the basic setting with binary point treatment and binary outcome, the method (i) estimates expected outcomes for treated and nontreated observations (e.g., individuals), (ii) estimates probabilities of receiving treatment for all observations, (iii) estimates a fluctuation parameter to inform how much to update the initial outcome estimates, (iv) updates initial outcome estimates, and (v) computes the average treatment effect and the standard error (SE) for statistical inference. 3) Causal forests: A forest-based method to estimate treatment effects with statistical inference (*105*). For a detailed explanation of random forests, see Table 2. 4) Double machine learning (DML): In the setting with binary treatment, the following steps are performed, for the case of twofold cross-fitting: (i) The method randomly splits the data into two sets; (ii) with the first set, predicts the outcome on the basis of covariates using ML; (iii) with the first set, predicts the treatment on the basis of covariates using ML; (iv) regresses the outcome residuals obtained from (ii) on the treatment residuals (iii) to obtain a model; (v) with the second set, uses the model derived from (iv) to obtain the estimate of the treatment effect; (vi) repeats the same procedure for the second set; and (vii) averages the treatment estimators obtained from the two sets for the final estimation (*106*).
Causal structural learning 1) Constraint-based algorithms: First learn an undirected graph and then determine orientation. Examples include the following: (i) stable PC algorithm (*142*) conducts numerous conditional independence tests to learn the DAG structure and solves the issue of order dependency in the classical PC algorithm (*143*); (ii) fast causal inference (*144*) is an extension of the PC algorithm to account for possible latent variables, when the assumption of causal sufficiency may not be met, i.e., not all common causes are measured; (iii) max-min parent and children is a two-phase algorithm for learning the direct causes and effects of any variable in the network (*145*); and (iv) incremental association Markov blanket (IAMB) (*146*) uses the concept of a Markov blanket to reduce the number of conditional independence tests (for a particular variable, the Markov blanket is the smallest conditioning set that ensure the particular node is conditionally independent of all others in the graph). 2) Score-based algorithms: Search over DAGs and score each on the basis of a specified objective function. Examples include the following: (i) hill climbing: starts with an empty graph and iteratively performs local additions, deletions, or reversal of edges to improve the graph’s score (*147*); (ii) Tabu search is an extension of hill climbing that attempts to help the algorithm escape local modes (*147*); and (iii) fast greedy equivalence search: adds and removes a polynomial number of edges to search over the space of equivalence classes of DAGs (*148*). 3) Hybrid approaches: Combine constraint-based and score-based. Example: Max-min hill climbing (*149*) uses constraint-based max-min parent and children to build the skeleton of the DAG, followed by score-based hill climbing to determine orientation.

### ML for causal inference 1: Counterfactual prediction

According to Hernán *et al*. (*6*), “Counterfactual prediction is using data to predict certain features of the world as if the world had been different”. A useful distinction to arrive at an answer if prediction or counterfactual prediction is sought is to ask whether the goal is “to explain or to predict?” (*100*), although we concede that, in some cases, we may be able to estimate the magnitude of a causal effect but not explain it (e.g., in a trial) (*6*). Causal questions in the potential outcomes framework (for counterfactual prediction) can be answered with traditional methods, e.g., regression and more advanced methods, such as marginal structural models (*6*). However, over the last years, ML for causal inference has grown substantially and is particularly helpful if embedded in frameworks of causal and statistical inference (*101*).

#### Common ML approaches for counterfactual prediction

For counterfactual prediction, ML can be used to analyze large sets of observational data by setting up the data in a way that causal assumptions are met and then use predictive ML approaches to answer causal questions (*102*). Other ML approaches are well suited to address causal questions if the data are set up properly. Examples are BART (*103*), TMLE (*104*), and Random Forests (*105*). Another ML approach to answer causal questions is double machine learning (DML), which learns the average treatment effect and the average treatment effect on the treated in high-dimensional settings (*106*). DML takes advantage of prediction accuracy of ML methods while providing unbiased and root *n*-consistent estimator with valid statistical inference through sample-splitting and cross-fitting. Reinforcement learning can also be conceptualized as a method to approach causal inference in research contexts where data generation (A/B testing) is possible. An overview of common ML approaches for causal inference using the potential outcomes framework is given in Table 3.

In some cases, the causal structure of (part of) the variables will be known, for example, if the variables are both linked and temporally ordered or follow another causal logic (e.g., researcher manipulation of the independent variable or another exogeneous cause). In the data science fields, one would speak of structured high-dimensional input. In these cases, we can use graphs for ML to incorporate this causal knowledge and extend predictive algorithms such as LASSO or neural networks to, for example, fussed LASSO or convolutional neural networks to reflect the causal structure.

##### Detailed description: BART

BART is presented here as an interesting approach to tackle counterfactual prediction, as it combines typical methods for causal inference, for example, propensity score weighting to balance the probability of treatment assignment and confounder adjustment to calculate counterfactuals that are used to estimate effects of treatment, so many of the necessary researcher decisions are made explicit. In more detail, BART is a sum-of-trees model predicting outcomes as the sum of a collection of individual regression tree fits and an additive Gaussian error term. Each regression tree iteratively applies splitting rules to partition the data into nonoverlapping subsets, aiming at minimizing the variance within each subset (*55*, *103*). As single trees overemphasize interactions and struggle to identify true linear relationships, subsequent trees are fit on the residual-predicted values for identified subsets (*103*). To avoid overfitting, BART introduces regularization priors for tree size (i.e., the number of subsets/terminal nodes) and shrinkage (i.e., a factor leveling means in subsets). However, the number of trees remains as a tuning parameter for BART models (*107*). A more detailed description of the underlying Bayesian backfitting algorithm can be found elsewhere (*103*). Implementations of BART exist for both regression and classification settings. Unlike common tree-based ML approaches such as Random Forests or boosting, regularization priors convey flexible tendencies (e.g., toward small trees) rather than fixed parameters identified by computationally heavy grid searches. Priors are further applicable to high-dimensional data and smoothing regression functions (*55*). Performance was shown to compete or exceed common approaches such as boosting, neural networks, or Random Forests. However, especially for binary outcomes, cross-validating BART models to choose regularization priors is advantageous (*55*, *103*). Besides computational benefits, BART is applicable to a wide variety of research foci and outcomes (e.g., survival and multinomial logistic regressions) and especially well equipped for causal inference tasks as modeling complex response surfaces and controlling for confounding do not rely on parametric assumptions (*55*, *107*). Resulting posterior distributions allow to estimate individual average treatment or heterogeneous causal effects. Moreover, the underlying likelihood framework delivers probabilistic statements about the outcome including credibility intervals, whereas identifying and quantifying the effect of individual variables on the outcome is more complicated (*107*). Recent adaptations and implementations of BART further allow modeling and including scores for probabilities of treatment and simulating treatment effects in the presence of unobserved confounding. In addition to that, procedures to control for the lack of common support are available (*55*).

Common research purposes in the social and health sciences for causal inference, usually addressed with the potential outcomes framework, are (i) the evaluation of potential causes of adverse social or health outcomes, (ii) the assessment of comparative treatment effectiveness, (iii) the identification of heterogeneous treatment effects, and (iv) the assessment and possible removal of bias in the statistical analysis.

#### Evaluation of potential causes of (adverse) social or health outcomes

With the aim to assess the effect of candidate causes, research designs may be set up to evaluate the ability of a predictor to causally influence an outcome in the context of controlling for confounders. One study tested the effects of fruit/vegetable density in nutrition of mothers-to-be on adverse pregnancy and birth outcomes, showing that TMLE outperformed traditional modeling by finding small effects and giving more precise estimates (*108*). Taking this question further, the follow-up study showed with doubly robust ML that the protective effects of fruit and vegetable density on risk of preeclampsia were modified by BMI of the soon-to-be mothers, with the protective effects strengthening with increasing BMI between the scores 20 and 30 and the effect plateauing for soon-to-be mothers with BMIs of 30 and higher (*109*). Applying a causal perspective even helped to solve the obesity paradox in critically ill patients (*110*). As an example from social mobility research, parental and individual socioeconomic determinants of income were compared with a longitudinal perspective with regression trees (*111*).

#### Assessment of comparative treatment effectiveness

Here, researchers may evaluate which of several treatments (e.g., intervention versus control or care-as-usual) is most effective in changing the health outcome. A selective overview of more clinical applications in health services research can be found in (*112*). As there are similarities in evaluating the “treatment” also in policy evaluations, we refer to an overview of developments on ML-based estimation of average treatment effects in economics in (*113*). One study tested the targeted prescription of cognitive-behavioral therapy versus person centered by estimated therapy outcome had they been assigned to the other treatment (=counterfactual outcome) (*114*).

#### Identification of heterogeneous treatment effects

In contexts with randomized treatment assignment, or a temporal research design to assess the effect of a newly introduced or changed policy, counterfactual prediction to estimate average treatment effects is straightforward. In addition, researchers may want to identify and describe subgroups who respond differently to treatment, that is, to explore heterogeneous treatment effects. Analyses of heterogeneous treatment effects can give answers to questions particularly prominent in public health research: What works best for whom, and when? For the technical explanations, see literature on estimating treatment effect heterogeneity (*115*). Random Forests have been developed to detect heterogeneous treatment effects (*105*). DML has also been used to estimate heterogeneous treatment effects (*106*).

While reanalysis of clinical trials is tempting to better understand possible heterogeneity in responding to an intervention or medical treatment, still, some caution is warranted: Heterogeneous treatment effects to reanalyze failed trials can be considered problematic and a form of p-hacking, as trials available for reanalysis usually have been designed to yield average effects (*116*). In general, we recommend that most of these approaches of using ML to identify heterogeneous treatment effects be used for hypothesis generation, with specific subgroup effects verified in an external population.

#### Assessment and removal of bias

Last, a number of interesting ML applications have been developed to quantify and address potential bias in analyses aiming at causal inference. In the absence of ignorability (no unmeasured confounders), sensitivity to unmeasured confounding may severely limit the generalizability of the study findings. The “treatsens” package estimates the magnitude of an unmeasured confounder that would be necessary to nullify the association between a treatment and the outcome; however, domain knowledge is needed in this analysis (*56*). In contexts with limited (or improperly realized) randomization, unbalanced distributions of covariates may be biasing the findings. Here, BART can assess the lack of common support (*117*), and covariate prioritization versus matching can adjust for differential probability to receive treatment (*118*). An overview of different papers that use ML for inverse probability weighting and propensity score matching is given in (*102*). Particularly in aging research, we recommend systematically assessing bias coming from selective attrition and competing risk of death, e.g., with random survival forests (*119*) that have been applied in studies on dementia (*41*).

### ML for causal inference 2: Causal discovery or causal structural learning

In contrast to the framework of Hernán *et al.* (*6*), other approaches suggest that causal inference does not necessarily need counterfactual prediction (*120*). Although rarely used in the health and social sciences, learning causal structure from the data is particularly interesting in contexts where data generation (manipulation of treatment) is possible. Even in settings without possibility for treatment manipulation, however, such as with observational data, causal structural learning may elucidate causal research questions: What about putative causes that cannot be manipulated in the sense of randomly assigning the exposure in a (real or hypothetical) intervention? We argue that the absence of a hypothetical intervention should not limit us in estimating a causal effect, e.g., of sex, as assessing the effect size of a social factor is a prerequisite to improving our understanding of the phenomenon under study and in the development of targeted interventions (*121*). Distinct relationships of race and sex/gender to socioeconomic and behavioral indicators are able to give insights into societal conditions (*122*). Nonmanipulable variables such as race or sex/gender can be reconceptualized in a way that allows manipulation or some form of intervention, for example, using perceived race in vignette studies. Going beyond the potential outcomes framework, however, we argue that, in these cases where a hypothetical intervention is absent, we can capitalize on structural causal learning, i.e., using algorithms that are able to learn (and present) causal structure of variables from the data. A guide on methods for causal discovery in cohort data has recently been proposed (*123*). In the following, we will first give an overview to structural learning before moving to the more complex task of deriving causal inference from graphical structures, so-called causal structural learning.

#### Structural learning

We start by considering structure learning for undirected graphs, that is, learning the conditional independence structure across complex high-dimensional data. The graphical LASSO (*124*) is widely adopted in this setting and is based on an underlying Gaussian assumption (i.e., for continuous variables). Various extensions of the Gaussian graphical LASSO have been developed, including extensions to simultaneously learn and estimate the network structure of variables across groups (e.g., corresponding to distinct subpopulations or data collected under different conditions) and across space and/or time (e.g., longitudinal data), for mixed variables (i.e., measurements on both continuous and discrete variables) and for missing data. This is particularly interesting in contexts where data are sparse, that is, contain many empty cells, and the simultaneous processing of all available data would be computationally extremely costly. A recent study used latent Gaussian graphical models for mixed variables, that is, binary, continuous, and count variables to infer symptom associations in verbal autopsies (*125*), which may be helpful to arrive at more robust classifications of probable causes of death.

#### Causal structural learning

Causal structural learning extends structure learning by also inferring the direction of the edges in the graph. Constraint-based approaches proceed by first learning an undirected graph, representing the skeleton of the DAG, and then determining orientation. Alternatively, score-based approaches search directly over the space of DAGs and score each graph on the basis of a specified objective function; the massive number of DAGs, which grows super-exponentially with the number of nodes/variables, requires carefully constructed search algorithms and scores. Hybrid algorithms combine ideas from constraint-based and score-based approaches. Pearl (*120*) provides a thorough technical overview of causality, and recent reviews of causal structural learning are provided in (*126*, *127*). An empirical evaluation and comparison of causal structural learning algorithms under noisy data assumptions can be found in (*128*). In the application of causal structural learning, performance relies on appropriateness of underlying assumptions (different models rely on different assumptions), sample size has only a weak influence on performance (varied from a few hundred to 20,000), and sparser graphs are easier to estimate (*127*). The “BNLEARN” package in R implements a number of constraint-based and score-based algorithms for causal structural learning (*129*).

A constraint-based approach, specifically the incremental association Markov blanket algorithm in BNLEARN, has been used for the identification of risk factors (which are possible causes) of HIV status, testing, and knowledge in the demographic and health surveys in 29 sub-Saharan African countries (*130*). While the data are cross-sectional and no unmeasured confounding is assumed, the lack of sociobehavioral determinants of HIV status, but the presence of sociobehavioral determinants of HIV knowledge and testing, gives insights into the limits of survey data collection when determining spread of infectious diseases. Another study used Markov blankets and the score-based algorithm fast greedy equivalence search to identify, among other goals, constitutive factors of race and sex/gender (*122*). While sex/gender was predominantly linked with personality characteristics and behaviors, race was linked to systemic factors and behaviors indicating socioeconomic deprivation, consistent with the idea that racial categories are constructed within a system (“structural racism”) (*122*).

We concede that applications of these methods will need the on-boarding of social and health researchers by collaborators trained in these methods, as using structural causal learning means also that there must be efforts to understand and communicate findings. Using this method will generate more complex sets of results, for example, an increasing number of possibly competing graphs. With a strong emphasis on the potential outcomes framework in virtually all disciplines in the social and health sciences, other methods for causal inference (and their potential benefits) remain widely unknown at this point in time. Having discussed ML tasks for description, prediction, and causal inference, we will elaborate on the most common ML performance metrics in the following section.

## ML PERFORMANCE METRICS

After the mapping of ML methods to the research goals of description, prediction, and causal inference, we can compare performance across different ML models and/or evaluate the use of ML compared to more traditional statistical methods. For this purpose, Table 4 gives a nonexhaustive list of common performance metrics to evaluate ML models. Evaluating the quality and performance of the model, ML models offer less straightforward solutions compared to more traditional modeling in the social and health sciences, where, for instance in economics, conditions of normality, consistency, and efficiency need to be met and can be tested rather straightforwardly (*113*). While resulting models can be evaluated within the ML framework, that is, a range of models is compared and the performance metrics are used to choose one model over the other(s), it is still state of the art to compare models from ML methods to those based on more traditional statistics such as logistic regressions. Some performance metrics (e.g., related to classification: sensitivity and specificity) are applicable to both ML and non-ML methods and can be used across frameworks.

**Table 4.** Overview and nontechnical description of common performance metrics to evaluate ML models.

**Indicator**	**Explanation and example**
**Unsupervised learning**	
**Specific to clustering**	
Adjusted rand index	Measure for the similarity of two data clusterings; related to accuracy but for unlabeled data.
Mutual information	Measure of mutual dependence, evaluates difference of joint distribution of two sets of variables to the product of their respective marginal distributions.
Calinski-Harabasz	Criterion to determine the “correct” number of clusters, several related criteria, e.g., implemented in the kml package to cluster longitudinal data.
Dunn index	Validates clustering solutions
**Specific to dimensionality reduction**	
Reconstruction error	Measure of the distance (e.g., Euclidean for continuous data) between the observed data and the “reconstructed data” from the inferred low-dimensional latent variables.
**Supervised learning**	
Variable importance	Variable importance quantifies the individual contribution of a variable to the classification or regression performance. Several implementations exist. For tree-based models such as Random Forests, variable importance is often modeled as the sum of improvements gained by using the variable in a split, averaged across all trees (*4*). In classification and regression, usually, the 5 to 10 most important variables can be meaningfully interpreted.
	As importance is not easily comparable to traditional statistics metrics, researchers may compare variable importance across multiple models and add a random variable as benchmark in consideration of statistical versus clinical (applied) importance.
**Specific to regression**	
Accuracy	Rate of correctly classified instances over all predictions (=true positives + true negatives/ true positives + true negatives + false positives + false negatives).
	Good measure in (close to) balanced data, i.e., outcome classes of similar rate.
	Do not use in imbalanced data.
Balanced accuracy	Arithmetic mean of sensitivity and specificity (see below); average of the proportion of correctly classified cases of each class individually, relevant in imbalanced classes.
Root mean square error (RMSE)	Average squared difference between the target value and the predicted value by the model. Penalizes large errors.
Mean square error	Preferred metric for regression tasks.
	Average of the square of the difference between original and predicted values.
Rank order	To evaluate importance of predictors across models or across samples, e.g., to predict dementia (*41*).
Subdistribution hazard ratios	To evaluate single predictors in regressions, e.g., to predict dementia (*41*), can be reported with confidence intervals.
Mean absolute error	Average of the absolute difference between original and predicted values.
**Specific to classification**	
Area under the curve (AUC)	For the classification of dichotomous outcomes, this metric specifies the area under the ROC curve, see below, with a range between 0 and 1. The larger the value, the better the model.
Logarithmic loss/log loss	Measures performance of a classification model, which provides predicted class probabilities. The log loss will get larger when the deviation of the predicted probability from the actual class value increases. Penalizes false classification. Works well in multiclass classifications (see multinomial logarithmic loss). Minimizing log loss will result in greater accuracy.
Mean absolute percentage error (MAPE)	Measure of prediction accuracy. It usually expresses the accuracy as a ratio.
Precision	In classification with two classes, relevant when the cost of false positives is high.
	The proportion of correctly classified cases of all classified cases (e.g., subjects), percentage, = true positives / (true positives + false positives).
Percentage correctly classified	Useful for multiclass classification (with more than two categories), easily interpretable.
Recall or sensitivity or true-positive rate (TPR)	In classification, relevant when the cost of false negatives is high.
	The rate of correctly classified cases of all actual positive cases [true positives by (true positives + false negatives)].
	Correctly classified cases in different strata, e.g., stratum with highest risk, with medium risk, and with lowest risk, e.g., to predict veteran suicide (*71*).
Receiver operating characteristic (ROC) curve	In classification with two classes.
	Allows to visualize the trade-off between the true-positive rate against the false-positive rate. The ROC curve shows the performance of a classification model at all classification thresholds.
Area under the precision-recall curve	In binary classification.
	Shows the trade-off between the true-positive rate (recall/sensitivity) against the positive predictive value (precision) at all classification thresholds. It is relevant in imbalanced datasets with a low prevalence of positives, where true positives need to be emphasized.
	A larger value reflects a better model.
Specificity	In classification.
	Proportion of correctly classified negatives, the rate of true negatives.
	Equal to one false-positive rate.
F1 score	Combines both precision and recall, i.e., a good F1 score would mean both false positives and false negatives are low.
**Specific to causal inference**	
**Counterfactual prediction**	
Average treatment effect	Difference in outcomes if all observations had received treatment compared to the scenario if no observations had received treatment.
Absolute bias estimate	Sensitivity to unmeasured confounding; in treatsens, the estimate of the unmeasured confounder to render the effect of the putative cause to zero [“Coeff. on U” in (*56*)].
Point estimate, difference in proportions	Effect estimate comparing two treatments, e.g., in BART and other algorithms used for causal inference; see, e.g., (*118*); can be average or heterogeneous treatment effect. Reported with 95% confidence intervals.
Adjusted risk difference	Evaluation of effect of candidate cause; linked average treatment effect in TMLE; see, e.g., (*108*).
	Reported with 95% confidence intervals.
SE	Gives confidence of treatment effect estimate. Calculated by, e.g., TMLE, for statistical inference
**Causal discovery**	
Structural hamming distance (SHD)	Metric for comparing two graphs by the number of changes required to transform one graph to another, widely used but is biased toward sensitivity of identifying edges over specificity.
Balanced scoring function	Metric for comparing two graphs that address the bias in SHD by taking into account all elements of the confusion matrix.
Metrics from classification	Metrics such as AUC or TPR specific to classification (see above) can be used to compare graphs, i.e., true edge rate = true-positive rate, false edge count = false positive, and missed edge rate = false negative.

## LOOKING FORWARD

Common research questions in the social and health sciences can be mapped to appropriate ML approaches, using distinctions between the research purposes of description, prediction, and causal inference. This review provides a new mapping for these research purposes that will enable the more systematic use of ML in the social and health sciences. The review as such is the most comprehensive overview of applications of ML in the social and health sciences to date. However, we could not cover in this review the challenges of the use of ML regarding research infrastructure and computational requirements, as well as issues of privacy and data protection. We also only marginally touched upon ML algorithms that have been developed for more automatic and faster processing of big data, such as natural language processing for speech or text recognition and sentiment analysis, and we provided only selective examples related to the analysis of omics data. Applications that use SNA for tourism or marketing purposes are discussed elsewhere (*53*).

ML approaches have potential to considerably improve empirical analysis if thoughtfully applied to relevant problems, ideally through collaborations between researchers trained in the social or health sciences and methodologically trained researchers. We hope this review will systematize and advance the uptake of the recently developed ML methods in social and health research.
